# Incidence of medically attended influenza and influenza virus infections confirmed by serology in Ningbo City from 2017–2018 to 2019–2020

**DOI:** 10.1111/irv.12935

**Published:** 2022-01-05

**Authors:** Cuiling Xu, Xuying Lao, Hongyu Li, Libo Dong, Shumei Zou, Yi Chen, Yongquan Gu, Yueqin Zhu, Pingfeng Xuan, Weijuan Huang, Dayan Wang, Bo Yi

**Affiliations:** ^1^ Chinese National Influenza Center, National Institute for Viral Disease Control and Prevention, Collaboration Innovation Center for Diagnosis and Treatment of Infectious Diseases, Chinese Center for Disease Control and Prevention, Key Laboratory for Medical Virology National Health Commission Beijing P.R. China; ^2^ Ningbo Municipal Center for Disease Prevention and Control Ningbo P.R. China; ^3^ Laboratory of Microbiology Gansu Provincial Center for Disease Control and Prevention Lanzhou P.R. China; ^4^ Yuyao Municipal Center for Disease Prevention and Control Ningbo P.R. China; ^5^ Lanjiang Street Community Health Service Center Ningbo P.R. China; ^6^ Yangming Street Community Health Service Center Ningbo P.R. China

**Keywords:** acute respiratory illness, burden of disease, China, incidence rates, infection, influenza

## Abstract

**Objectives:**

In mainland China, the disease burden of influenza is not yet fully understood. Based on population‐based data, we aimed to estimate incidence rates of medically attended influenza and influenza virus infections in Ningbo City.

**Methods:**

We used data for outpatient acute respiratory illness (OARI) from a platform covering all health and medical institutes in Yingzhou District, Ningbo City. We applied generalized additive regression models to estimate influenza‐associated excess incidence rate of OARI by age. We recruited local residents aged ≥60 years in the autumn of 2019 and conducted follow‐up nearly 9 months later. Every survey, the sera were collected for testing hemagglutination inhibition antibody.

**Results:**

From 2017–2018 to 2019–2020, the annual average of influenza‐associated incidence rate of OARI in all ages was 10.9%. The influenza‐associated incidence rate of OARI was the highest in 2017–2018 (16.9%) and the lowest in 2019–2020 (4.8%). Regularly, influenza‐associated incidence rates of OARI were the highest in children aged 5–14 years (range: 44.1–77.6%) and 0–4 years (range: 8.3–46.6%). The annual average of excess OARI incidence rate in all ages was the highest for influenza B/Yamagata (3.9%). The overall incidence rate of influenza infections indicated by serology in elderly people was 21% during the winter season of 2019–2020.

**Conclusions:**

We identified substantial outpatient influenza burden in all ages in Ningbo. Our cohort study limited in elderly people found that this age group had a high risk of seasonal influenza infections. Our study informs the importance of increasing influenza vaccine coverage in high‐risk population including elderly people.

## 1 INTRODUCTION

Influenza viruses cause a substantial burden on morbidity and mortality worldwide.^1^, ^2^ In the developed countries, the disease burden associated with influenza has been increasingly understood.^3^, ^4^ Mortality, rate of hospitalization, incidence of cases seeking medical care, and incidence of symptomatic and asymptomatic infection with influenza usually are used to understand the profile of influenza‐associated disease burden.^5^

China is a large country with a diversity of climatic zones. The previous studies have characterized seasonality patterns of influenza epidemics by type, subtype, or lineage that varied across the country.^6^, ^7^ The disease burden of influenza in China is not yet fully understood. Although hospital‐based influenza‐like illness (ILI) sentinel surveillance network has been built in mainland China to monitor influenza activity,^6^, ^7^ there is a challenge in estimating influenza‐associated outpatient disease burden for the surveillance network. The challenge may arise from difficulty in determining well‐defined catchment populations of surveillance sentinel hospitals and underrepresentation of sentinel hospitals as most of sentinel hospitals are ones at tertiary level and located in prefectural cities.^8^ Additionally, few data are available about disease burden of infections with seasonal influenza in elderly people at community level in mainland China.

Ningbo is an economically developed coastal city located in the southeast of China and has two peaks of influenza activity in November to February and July to September.^6^, ^7^ Since 2015, an Integrated Platform of Health and Medical Big Data (IPHMBD) covering all public and private health and medical institutes in Ningbo City has been established.^9^ The platform aims to improve the management of health and medical big data and exploit fully these data to provide evidence for decision making of public health and medical care. The platform in Yingzhou District of the city is the first one with similar function that was completed and put into use in China. The objective of this study was to estimate influenza‐associated incidence rate of outpatient acute respiratory illness (OARI) based on the population‐based data platform. Additionally, we established a community‐based cohort for people aged ≥60 years to qualify the incidence rate of seasonal influenza infections indicated by serology during the wintertime influenza season of 2019–2020 in Yuyao City, Ningbo City. Understanding influenza‐associated burden in the outpatient setting and in the community is important for improving the strategy of influenza vaccine recommendation in mainland China.

## 2 METHODS

### 2.1 Data sources

#### 2.1.1 The IPHMBD

We used data from the IPHMBD in Yingzhou District, Ningbo City. The platform collects data from hospital information system of the all public and private medical and health institutes located in Yingzhou District for the purpose of disease surveillance and control.^9^ The outpatient and inpatient data in these medical and health institutes can be transferred in real time to the platform. The records with the diagnosis of OARI related to influenza under the International Classification of Diseases, Tenth Revision (ICD‐10) code were extracted. The ICD‐10 codes related to acute respiratory illness include J11.101, J06.900, J06.901, J06.903, J02.801, J02.900, J03.900, J00.x02, and J00.x03. The number of OARI was compiled by the designated data manager of IPHMBD and then was offered to us. The data we received did not include any identification information of patients.

To remove the repeated visits of patients due to the same episode of illness, we define an OARI with the time interval of 7 days or less between two consecutive medical visits as the same episode of influenza‐related respiratory illness. An OARI with the time interval of more than 7 days since the past medical visit is defined as a new episode of influenza‐related respiratory illness. For those repeated visits due to the same episode of illness within the time interval of 7 days, we only counted them as one episode of influenza‐related illness. Incidence rates of OARI in 0–4, 5–14, 15–14, 25–59, and ≥60 years and all ages combined were calculated by the number of OARI divided by the size of resident population in these age groups of Yingzhou District.

#### 2.1.2 Influenza surveillance data

We used weekly influenza laboratory surveillance data collected by a laboratory and two sentinel hospitals based in Ningbo City as part of the Chinese Influenza Sentinel Surveillance Network from October 2017 through September 2020. The two sentinel hospitals for influenza surveillance are at tertiary level: One is a comprehensive hospital, and the other is a children hospital. According to the National Influenza Surveillance Guideline, each sentinel hospital reports weekly count of outpatients with ILI by age group (ILI is defined as body temperature ≥38°C with either cough or sore throat) to a web‐based national information system. Each sentinel hospital is also required to collect 20 or more pharyngeal swab specimens from ILI outpatients per week. The respiratory specimens are transported to the network laboratory for influenza detection. These sentinel hospitals and the network laboratory in Ningbo City conduct year‐round influenza surveillance.

Respiratory specimens were tested by polymerase chain reaction (PCR) or were inoculated into Madin–Darby canine kidney cells and/or chicken embryos for virus isolation and hemagglutination inhibition (HI) to identify subtypes of influenza A and lineages of influenza B in the network laboratories. ILI specimens that tested positive for influenza virus were sent to Influenza Reference Center based in Zhejiang Provincial Center for Disease Control and Prevention (CDC) for confirmation.

#### 2.1.3 Serological cohort study

Our cohort study was conducted in Lanjiang Subdistrict of Yuyao City, Ningbo City. Every year, local residents aged ≥60 years were provided with a free physical examination by community health service centers in this city. During the period of their physical examination, local residents aged ≥60 years were recruited by healthcare workers working at Lanjiang Community Health Service Center to participate in our study via face‐to‐face invitation from mid‐October to mid‐November 2019 (pre‐season survey). When these elderly people agreed to participate, they were asked to complete a questionnaire including demographics and underlying medical conditions. Serum samples were collected from the participants by trained nurses. The elderly participants were followed up from mid‐June to early‐August 2020 (post‐season survey). Seasonal influenza vaccination information about the participants during the 2019–2020 influenza season was collected by searching their resident identification code in IPHMD.

### 2.2 Laboratory test

The paired sera were tested in parallel by HI assays using five representative circulating strains for each of the seasonal influenza A subtypes and influenza B lineages in 2019–2020 influenza season. Five circulating representative strains were used for the serological test, including A/Brisbane/02/2018 (H1N1), A/Singapore/INFIMH‐16‐0019/2016 (H3N2), B/Phuket/3073/2013 (B/Yamagata), B/Colorado/06/2017(B/Victoria), and B/Sichuan‐Gaoxin/531/2018 (B/Victoria). In 2019–2020 influenza season, B/Victoria lineage viruses with different genetic characteristics circulated concurrently in China. B/Sichuan‐Gaoxin/531/2018‐like virus with a deletion of three amino acids in hemagglutinin and B/Colorado/06/2017‐like virus with a deletion of two amino acids in hemagglutinin were selected for the serological test. These influenza strains were provided by National Influenza Center, China CDC. Influenza B viruses for HI assay were treated with ether prior to use to increase sensitivity for the HI assay.^10^

The HI assay was carried out according to standard methods described in the World Health Organization (WHO) guideline.^11^ Specially, all hemagglutination assays to determine standardized antigen for HI test were performed using guinea pig red blood cells in the presence of 20‐nM oseltamivir carboxylate, which is added to circumvent the NA‐mediated binding of H3N2 viruses to the red blood cells. HI assays of H3N2 virus isolates are carried out in the presence of 20‐nM oseltamivir carboxylate.^12^, ^13^ Sera were tested in serial doubling dilutions from an initial dilution of 1:10 to endpoint dilution of 1:1280 by HI assays using standard methods. Antibody titers <1:10 were imputed as 1:5, and antibody titers ≥1:1280 were imputed as 1:1280.

### 2.3 Ethics

Written consent was obtained from all participants aged ≥60 years in the serological cohort study. The protocol of the cohort study was approved by the Institutional Review Board of Institute for Viral Disease Control and Prevention, Chinese Center for Disease Control and Prevention.

### 2.4 Statistical analysis

#### 2.4.1 Estimating influenza‐associated excess outpatient respiratory incidence rates

We applied a generalized additive model to derive the estimates of influenza‐associated excess incidence rates of OARI between 2017–2018 and 2019–2020 for Yingzhou District, Ningbo City. The generalized additive model was chosen to reflect the assumption that increases in influenza activity would lead to corresponding additive increases in incidence rates of OARI approximately with a normal distribution.^14^ Incidence rates of OARI in 0–4, 5–14, 15–24, 25–59, and ≥60 years and all ages combined were used as the dependent variables in the models and regressed against the proxies for influenza activity. We used percentages of ILI specimens tested positive for influenza by subtype/lineages (or called positive rate) as the influenza virus activity proxies. The cubic smooth spline functions were used to control the effects of calendar week and absolute humidity for linear and non‐linear changes in incidence rates of OARI. The presence or absence of Spring Festival and containment for COVID‐19 after Spring Festival in 2020 were included as covariates in the regression models. Given the possible delay between climatic factors and onset of influenza illness, we specified a lag of 1 week between absolute humidity and incidence rates of OARI.

The influenza‐associated excess incidence rates of OARI were estimated by subtracting the predicted incidence rate assuming that influenza activity for a specific type or subtype to zero from the predicted incidence rate from the model based on the reported weekly influenza activity.^14^ The influenza‐associated excess incidence rates of OARI was estimated as the number of influenza‐associated excess respiratory illness seeking outpatient consultants per 100 population. The 95% confidence intervals (CIs) for excess incidence rates were estimated with a bootstrap approach. We estimate the cumulative incidence rates of influenza‐associated excess OARI in each surveillance year, influenza wintertime season, and summertime season during the study period. A surveillance year was defined as a year from October of each year to September of next year. As it has been reported that Ningbo City located in Chinese midlatitude provinces could experience semiannual influenza activity, we divided each surveillance year into influenza wintertime season and summertime season, which were defined as the period between October of each year and March of next year and between April and September of each year, respectively.

#### 2.4.2 Estimating incidence rates of influenza virus infections confirmed by serology

Infection with seasonal influenza viruses was defined as a fourfold or greater rise in antibody titers between pre‐season and post‐season paired sera. We used Poisson regression model to estimate the incidence rates of influenza virus infections indicated by serology in people aged ≥60 years or older and the factors associated with influenza virus infections. We compared medians of pre‐season HI antibody titers between elderly people without receipt of seasonal influenza vaccine in 2019–2020 infected and not infected with seasonal influenza viruses, using Wilcoxon signed‐rank test after log transformation.

## 3 RESULTS

### 3.1 Surveillance data of influenza virus and OARI

**FIGURE 1:**
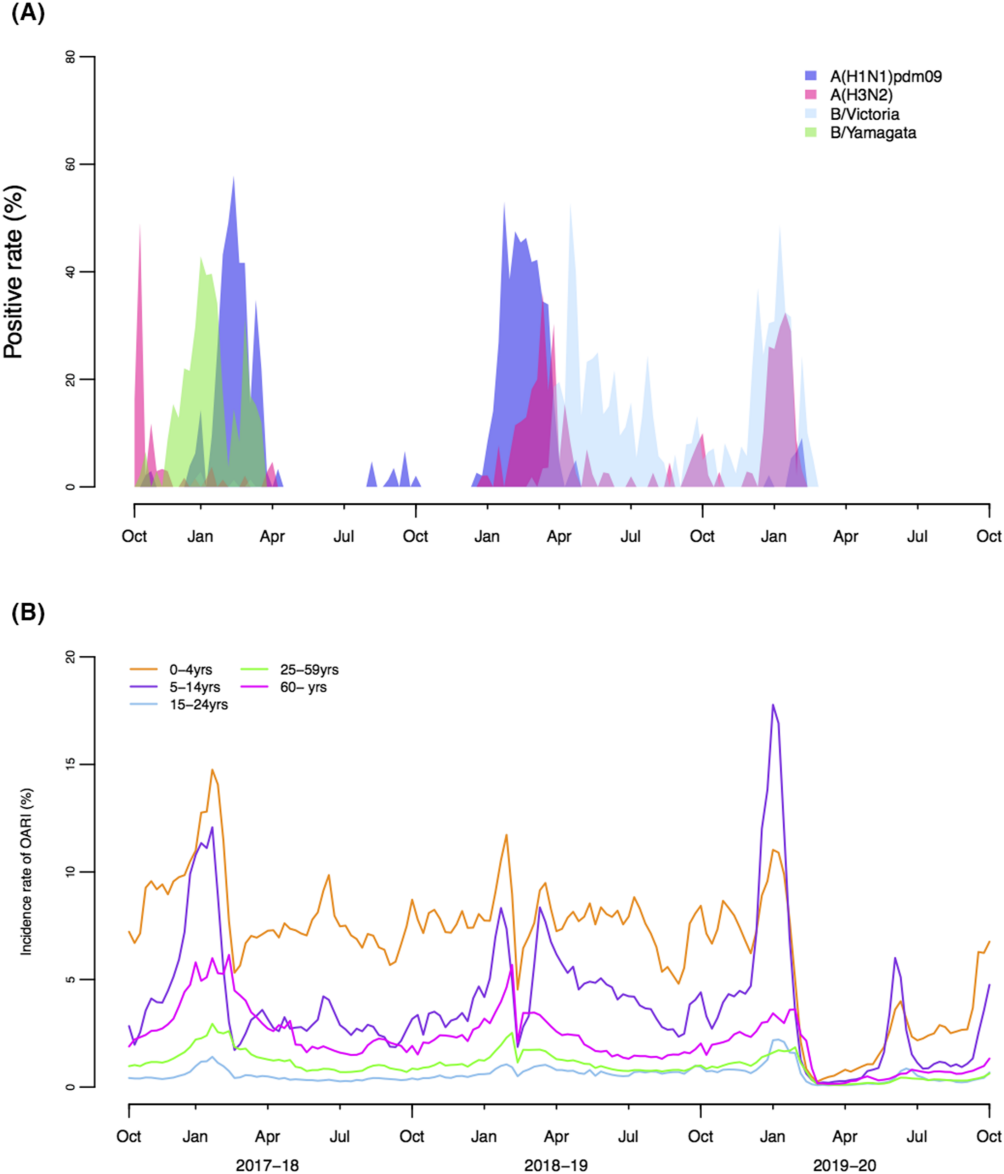
(A) Weekly percentages of influenza‐like illness specimens testing positive (positive rates) for influenza by subtype/lineage in Ningbo City, from 2017–2018 to 2019–2020. (B) Weekly age‐specific incidence rates of outpatient acute respiratory illness (OARI) by age group in Yingzhou District, Ningbo City, from 2017–2018 to 2019–2020. The age‐specific incidence rates of OARI were calculated by the number of OARI in Yingzhou District obtained from the Integrated Platform of Health and Medical Big Data divided by the population size by age group

From 2017–2018 to 2019–2020, there were influenza epidemic waves in Ningbo City in the winter and spring of each year, whereas there was only a weak summertime epidemic of B/Victoria that peaked in early‐April 2019. During the study period of 3 years, influenza A (H1N1)pdm09, A(H3N2), and B/Victoria virus circulated alternately and cause two to three major epidemics of different subtypes or lineages each year, whereas only an epidemic of influenza B/Yamagata occurred in the winter of 2017–2018 (Figure 1A). The epidemics in the winter of 2019–2020 ended abruptly in mid‐February 2020.

A total of 3,258,019 episodes of acute respiratory illness were reported in the outpatient setting of the public or private hospitals or clinics in Yingzhou District from 2017–2018 to 2019–2020. The overall numbers of OARI episodes in 2017–2018 and 2018–2019 (1,235,865 and 1,250,553, respectively) were similar, and much more than that in 2019–2020 (771,601). Totally, the annual average of incidence rates of OARI was 82% in all ages from 2017–2018 to 2019–2020. The annual average of incidence rate of OARI in children aged 0–4 years (348/100 persons) was the highest, followed by children aged 5–14 years (204/100 persons). The annual average of incidence rate of OARI in persons aged 15–24 years (31/100 person) was the lowest (Figure 1B).

### 3.2 Incidence rates of influenza‐associated excess outpatient respiratory illness

**FIGURE 2:**
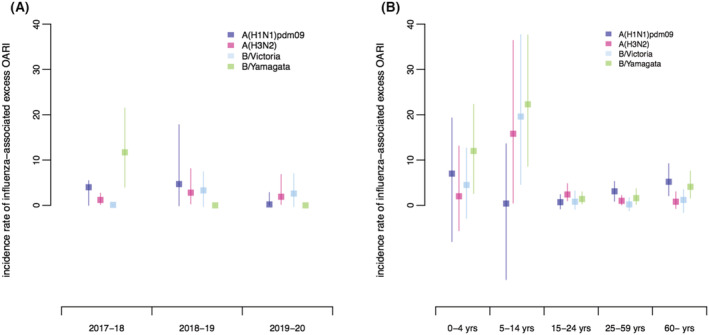
(A) The annual incidence rates of influenza‐associated excess outpatient acute respiratory illness (OARI) by age group in Yingzhou District, Ningbo City, from 2017–2018 to 2019–2020. (B) The annual incidence rates of influenza‐associated excess OARI by influenza subtype/lineage in Yingzhou District, Ningbo City, from 2017–2018 to 2019–2020

From 2017–2018 to 2019–2020, the annual average influenza‐associated excess OARI incidence rate in all ages was 10.9% (95% CI: 6.2–17.3%). The annual incidence rate of influenza‐associated excess OARI in all ages was the highest in 2017–2018 (16.9%, 95% CI: 8.5–28.4%), followed by 2018–2019 (10.9%, 95% CI: 3.8–21.9%), and then the lowest in 2019–2020 (4.8%, 95% CI: 1.0–12.7%). In each of three surveillance years, the annual age‐specific incidence rates of influenza‐associated excess OARI were the highest in children of 5–14 years old (range: 44.1–77.6%), followed by infants or children of 0–4 years (range: 8.3–46.6%), and the lowest in the age groups of 15–24 or 25–59 years old (range: 1.5–9.5%) (Table 1 and Figure 2A). The cumulative incidence rate of influenza‐associated excess OARI in all ages in the summertime season of 2017–2018 and 2019–2020 was 0% (95% CI: 0–0%) and 0.2% (95% CI: 0–0.6%), respectively, whereas the all‐age cumulative incidence rate in the summertime season of 2018–2019 was 3.9% (95% CI: 0.4–8.1%), which was approximately equivalent to one half of the rates in the wintertime season of this year.

**TABLE 1:** Incidence rates of seasonal influenza infections confirmed by serology by subtype and lineage during the 2019–2020 influenza season

Age groups	2017–2018			2018–2019			2019–2020		
	Oct–Mar	Apr–Sep	Total	Oct–Mar	Apr–Sep	Total	Oct–Mar	Apr–Sep	Total
0–4 years	46.1 (12.4–79.5)	0.5 (−0.7 to 1.8)	46.6 (12.2–80.1)	13.6 (−5.9 to 37.5)	7.6 (−2.5 to 20.8)	21.3 (−4.6 to 50.9)	8.3 (−1.80)	0 (0–0)	8.3 (−1.8 to 27.4)
5–14 years	77.5 (29.3–123.7)	0 (−1.6 to 1.2)	77.6 (29.2–123.9)	20.1 (−5.3 to 52.7)	35.6 (12.0–58.7)	55.7 (10.5–100.7)	41.1 (7.40)	0 (0–0)	41.1 (7.4–94.2)
15–24 years	6.4 (2.8–12.1)	0.1 (−0.1 to 0.2)	6.5 (2.8–12.2)	3.6 (1.2–8.1)	2.2 (−0.3 to 6.1)	5.8 (1.5–12.9)	3.4 (0.30)	0 (0–0)	3.4 (0.3–9.1)
25–59 years	9.2 (4.4–17.6)	0.2 (0–0.6)	9.5 (4.5–17.8)	5.8 (2.6–11.7)	0.8 (−1.4 to 3.3)	6.6 (2.3–13.8)	1.5 (−0.60)	0 (0–0)	1.5 (−0.6 to 5.2)
60 years	19.4 (9.6–33.8)	0.4 (0.1–1)	19.8 (10–34)	9.1 (3.6–19.5)	2.2 (−2 to 6.3)	11.3 (3.6–23.9)	2.7 (−1.80)	0 (0–0)	2.7 (−1.8 to 8.5)
All ages	16.7 (8.5–27.8)	0.2 (0–0.7)	16.9 (8.7–28.1)	7.0 (2.4–15.1)	3.9 (0.4–7.9)	10.9 (4.1–21)	4.8 (10)	0 (0–0)	4.8 (1.0–11.6)

*Note*: Data are the incidence rates of influenza‐associated excess outpatient respiratory illness (%) and 95% confidence interval.

During the study period, the annual average of excess OARI incidence rate in all ages was the highest for influenza B/Yamagata (3.9%, 95% CI: 1.5–7.2%), followed by that for influenza A(H1N1)pdm09 virus (3.0%, 95% CI: 0.1–6.8%), and the lowest for influenza A(H3N2) (2.0%, 95% CI: 0.3–5.4%) and B/Victoria virus (2.0%, 95% CI: −0.3 to 4.9%). As variation in the influenza activity by subtype and lineage occurred across three surveillance years, the all‐age annual incidence rate of influenza‐associated excess OARI attributed to different subtypes and lineages varied. The all‐age annual incidence rates of excess OARI ranged between 1.2% (95% CI: 0.3–2.6%) and 2.8% (95% CI: 0.4–7.9%) for A(H1N1)pdm09, between 0.3% (95% CI: 0–0.7%) and 4.7% (95% CI: 0.5–12.0%) for A(H3N2), between 0.1% (95% CI: 0–0.2%) and 3.3% (95% CI: −0.2 to 7.0%) for B/Victoria virus, and between 0% (95% CI: 0–0%) and 11.7% (95% CI: 3.9–21.2%) for B/Yamagata virus (Figure 2B).

### 3.3 Incidence rates of influenza virus infections indicated by serology

Our cohort study recruited 500 people aged ≥60 years from mid‐October to mid‐November 2019; 476 elderly people (95%) were followed up from mid‐June to early‐August 2020 and provided pre‐season and post‐season sera. The characteristics of subjects participating in pre‐season and post‐season surveys were similar (Table 2). Among participants followed up, 46% were male, and 51% were 70 years or older; 100% of participants followed up had tertiary education or above; and 45% of them were retired or did not have a temporary or stable job. Thirteen of the participants received seasonal influenza vaccine during the 2019–2020 influenza season (Table 2).

**TABLE 2:** Characteristics of participants aged 60 years or older completing pre‐season survey and post‐season survey across 2019–2020 winter influenza season in the cohort study in Yuyao City, Ningbo City

Characteristics	Pre‐season	Post‐season
Age (years)		
60–69	248 (49.6)	232 (48.7)
≥70	252 (50.4)	244 (51.3)
Gender (male)	235 (47.0)	219 (46.0)
Ethnicity (Han)	499 (99.8)	475 (99.8)
Working status		
Retired or not working	220 (44.0)	212 (44.5)
Having a job or as a farmer	279 (55.8)	263 (55.3)
Education		
Primary or below	464 (92.8)	441 (92.6)
Secondary	36 (7.2)	35 (7.4)
Tertiary or above	0 (0)	0 (0)
Self‐perceiving good health status	402 (80.4)	380 (79.8)
Influenza vaccination in 2019–2020^a^	13 (2.6)	13 (2.7)

^a^ The information on influenza vaccination of participants in 2019–2020 was obtained from the Integrated Platform of Health and Medical Big Data in Ningbo City.

Overall, we confirmed 115 seasonal influenza infections indicated by serology during the study period, including 13 influenza A(H1N1)pdm09, 23 A(H3N2), 82 B/Victoria, and 44 B/Yamagata. Of those infections, 37 (32%) had infections with two or more influenza subtypes or lineages in the same influenza season. The incidences of seasonal influenza viruses in people ≥60 years of age without uptake of seasonal influenza vaccine during the 2019–2020 influenza season were shown in Table 3. During the 2019–2020 influenza winter season, the overall incidence rate of influenza infections indicated by serology in people aged ≥60 years was 25% (95% CI: 21–30%). The incidence rate of infection with B/Victoria (18%; 95% CI: 14.3–22.0%) was the highest in people aged ≥60 years, followed by the incidence of infection with B/Yamagata (9.5%; 95% CI: 7–13%). The risks of infection with A(H1N1)pdm09 and H3N2 in people aged ≥60 years were 3–5%. The overall incidence rates of infection with influenza or subtype/lineage‐specific incidence rates were similar between people aged 60–69 years and those aged ≥70 years. Gender, education level, working status, and self‐perceiving health status were not associated with serological evidence of seasonal influenza infections.

**TABLE 3:** Incidence rates of seasonal influenza infections confirmed by serology by subtype and lineage during the 2018–2019 influenza season

Influenza subtypes/lineages	Incidence rates, % (95% CI)		
	60–69 years	≥70 years	Total
A(H1N1)pdm09	2.5 (1.1–5.6)	3.1 (1.5–6.5)	2.8 (1.6–4.8)
A(H3N2)	5.9 (3.5–9.9)	4.0 (2.1–7.7)	5.0 (3.3–7.5)
B/Victoria	18.4 (13.7–24.8)	16.9 (12.3–23.3)	17.7 (14.3–22.0)
B/Yamagata	10.9 (7.4–16.0)	8.0 (5.1–12.7)	9.5 (7.1–12.8)
Overall	26.0 (20.3–33.3)	23.6 (20.3–33.3)	24.8 (20.7–29.8)

Abbreviation: CI, confidence interval.

**FIGURE 3:**
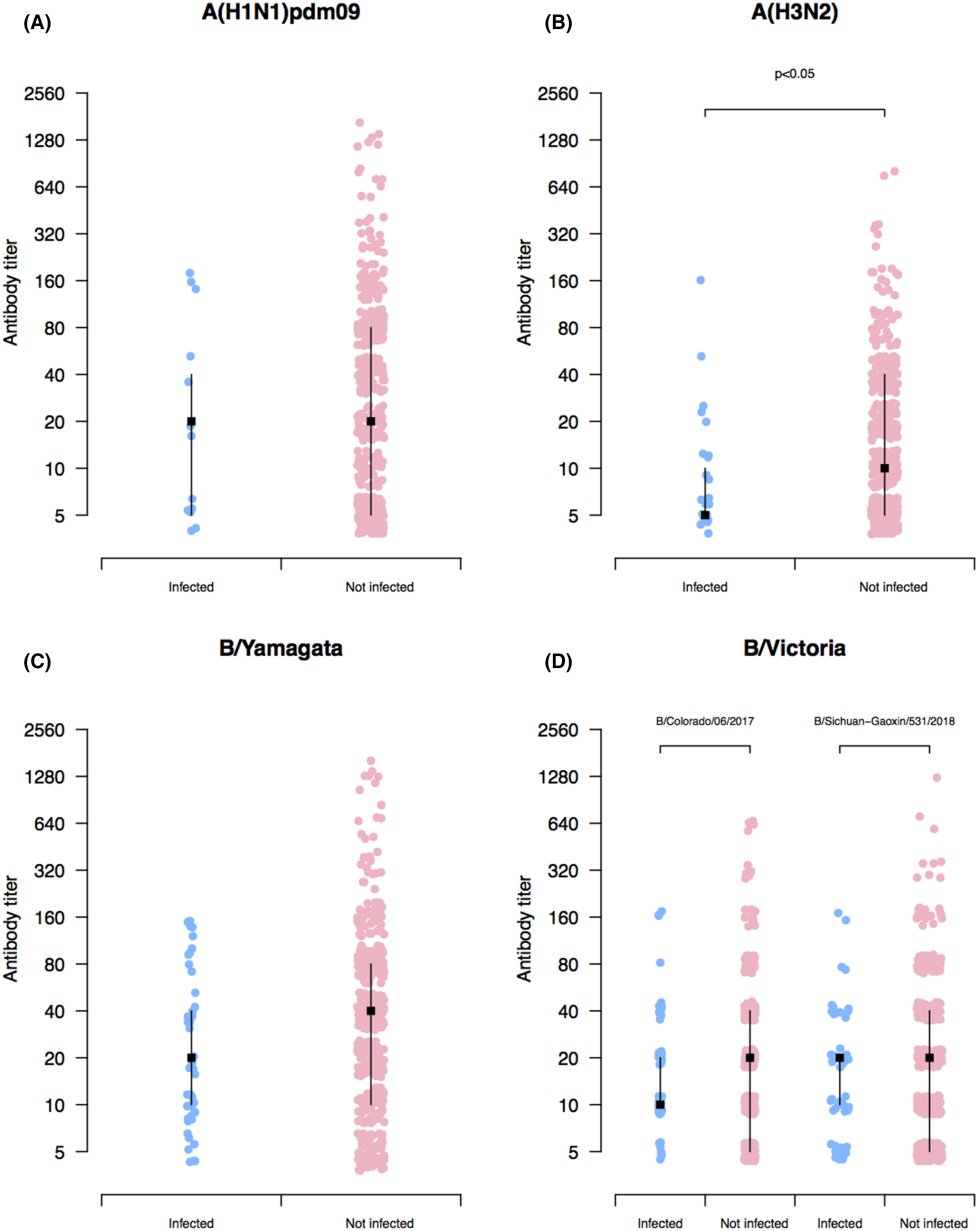
(A–D) Pre‐season hemagglutination inhibition (HI) antibody titer measurements (points), and medians and interquartile range against the five circulating representative strains of A(H1N1)pdm09, A(H3N2), B/Yamagata, and B/Victoria. B/Colorado/06/2017and B/Sichuan‐Gaoxin/531/2018 that were circulating representative strains of B/Victoria virus were selected for HI test. The *y* axis indicates the pre‐season HI antibody titer measurements. Two‐tailed Wilcoxon rank‐sum tests were used to compare pre‐season log‐transformed HI antibody titers between participants infected (blue dots) and not infected (pink dots) with each influenza strain

The pre‐season HI antibody titers against influenza A (H3N2) viruses in people aged ≥60 years not infected with the subtype virus were significantly higher than that in people aged ≥60 years infected with the subtype virus (P < 0.05) (Figure 3B). Pre‐season HI antibody levels against A(H1N1)pdm09, B/Victoria, and B/Yamagata were not significantly different between elderly people infected and not infected with these subtypes and lineages (Figure 3A,C,D).

## 4 DISCUSSIONS

The IPHMBD in Ningbo is the first information management and surveillance system for public health that incorporates all outpatient medical record and covers all residents of the jurisdiction in China. The system with high‐quality symptomatic surveillance data allows estimation of outpatient burden attributable to influenza via fitting a model in combination with virological surveillance data. We estimated the annual average of 10.9 outpatient visits per 100 persons (95% CI: 6.2–17.3) for acute respiratory illness attributable to influenza from 2017–2018 to 2019–2020. In 2019–2020, the overall outpatient burden attributable to influenza was the lowest (4.8%, 95% CI: 1.0–12.7%). It was probably a result of implementation of the containment measures against the emergency of COVID‐19. The containment measures, including prolonged Spring Festival holidays, social distancing, and mask wearing, could lead to a decline of occurrence of acute respiratory illness and an increase of self‐prescription. We think that the outpatient burden attributable to influenza in 2017–2018 and 2018–2019 (range: 10.9–16.9%) represents more the status during the interpandemic period.

Compared with a study in Germany from 2001–2002 to 2014–2015 (range: 0.7–8.9%) that uses acute respiratory illness as a case definition and estimated the burden by fitting a statistical model, there were higher estimates of outpatient burden in Ningbo in 2017–2018 and 2018–2019.^15^ One of the possible reasons for the higher outpatient burden in Ningbo could be that low influenza vaccine coverage leads to higher incidence rates of symptomatic and asymptomatic infection with seasonal influenza (oral communication). Additionally, as a result of high economic level, lower price, and high degree of convenience of health service in Ningbo, local residents tend to seek medical care to alleviate symptoms of respiratory illness. Compared with similar studies from China and other countries using ILI as case definition, the influenza‐associated outpatient burden in our study in 2017–2018 and 2018–2019 was higher.^8^, ^16^, ^17^, ^18^ Influenza virus infections lead to a wide range of clinical manifestations, variable from asymptomatic infection, mild symptoms, to severe pneumonia. ILI case definition may include the presence of fever (or another systemic symptom) in addition to one or more respiratory symptoms. OARI case definitions used in our study usually do not require an obligatory presence of fever or feverishness. Only a portion of all symptomatic influenza cases are captured by ILI case definitions.^19^ The surveillance systems using OARI case definitions are more suited to describe and capture the burden of disease of influenza.^15^

Similar to other studies, the age‐specific outpatient burden attributable to influenza among children of age 0–4 and 5–14 years was constantly the highest in each year.^8^, ^16^, ^17^, ^18^ Although the overall incidence rate of OARI in children aged 0–4 years was higher than that in children aged 5–14 years, the influenza‐associated outpatient burden for the two age groups presented the opposite situation. This could be associated with the a higher baseline level of OARI among children aged 0–4 years during the non‐epidemic period and a higher proportion of OARI that could be attributable to infection with other unmeasured relevant pathogens in this age group.

Noticeably, our study has identified not only substantial outpatient burden (range: 2.7–19.8%) in people aged ≥60 years but also high risks of infections with influenza indicated by serology in the age group. Although the wintertime influenza season in 2019–2020 ended earlier, the incidence rate of infection with influenza virus indicated by serology in people aged ≥60 years reached 25% (95% CI: 21–30%), which was slightly higher than that (21%; 95% CI: 17–25%) in adults 18–59 years in another serological cohort study conducted in Shanxi Province in 2018–2019.^20^ Contrary to other studies in other countries and China, our study indicated that the influenza‐associated outpatient burden in people aged ≥60 years was higher than those in adults under 60 years.^8^, ^15^, ^16^, ^17^, ^18^ The result needs to be verified for a longer time frame in the future study. The potential reason could be that elderly people are less likely to present fever or feverishness, and ILI case definition may capture less portion of outpatient illness attributable to influenza.

Our study estimated outpatient burden attributable to different influenza subtypes and lineages. Due to the annual variation of influenza subtype and lineage circulation, the annual outpatient burden attributable to different influenza subtypes and lineages varied. As influenza B/Yamagata had a strong impact on outpatient burden in 2017–2018 (11.7%; 95% CI: 3.9–21.2%), it had the most contribution to the annual average of outpatient burden across the 3 years. In 2017–2018 and 2018–2019, influenza A(H1N1)pdm09 led to higher burden in outpatient setting. Influenza A(H3N2) and B/Victoria virus had low outpatient burden in each of the 3 years.

Previous studies have identified that HI antibody titer measured was correlated with protection against influenza A and B virus infection.^21^, ^22^ Our study found that the higher pre‐season HI titer against influenza A(H3N2) indicated by serology was associated with the reduced risk of A(H3N2) infection indicated by serology among people aged ≥60 years. However, we did not identify the association of HI titers against influenza A(H1N1)pdm09, B/Victoria, and B/Yamagata with serologically confirmed infections with this influenza subtype and lineages. The absence of association of infection with influenza A(H1N1)pdm09 with HI titer in elderly participants may be attributable to low incidence rate of infections with influenza A(H1N1)pdm09 indicated by serology. However, here are limited data about the association of degree of protection against influenza B infection with specific HI titers in elderly people.^22^ One possible explanation is that HI titer may play more roles in protection against virologically confirmed infection with influenza B, but may be less protective of asymptomatic influenza infection that only can be confirmed by serology.

Our study showed that seasonal influenza vaccine uptake in elderly people in Ningbo was low. Only 2.6% of elderly people received seasonal influenza vaccine during the 2019–2020 influenza season. In Ningbo City, the influenza vaccine could be reimbursed by basic social medical insurance. Besides, free influenza vaccine has been recommended to high‐risk population including elderly people. However, the influenza vaccination rate is still far from satisfaction among elderly people, compared with high‐income countries. Several factors could affect low vaccination rate among elderly people. First, elderly people could have a misconception about safety and efficacy.^23^ Second, healthcare workers could offer little recommendation to elderly people for seasonal influenza vaccination.^24^ Finally, inadequate supply of seasonal influenza vaccine may hinder vaccine uptake.

Our study is subject to several limitations. First, due to the fact that our study was limited to two representative years with influenza seasonal cycle and 1 year when the seasonal pattern of influenza activity was interrupted by emergence of COVID‐19, we cannot estimate cumulative outpatient burden in a long time and cannot understand the pattern of outpatient burden by subtype/lineage. Second, we used resident population as an approximation of population covered by IPHMBD to estimate incidence; however, the number of OARI could capture some migrant population who stayed for less than 6 months in Yingzhou District or residents living in other districts of Ningbo. Some residents living in Yingzhou District could seek medical care to other districts of Ningbo, even to other cities. We think that the resident population is more suitable to our estimates, compared with data of other census tract. Additionally, we did not have age‐specific virological surveillance data on ILIs, and the use of aggregate surveillance data as the proxy of influenza activity in our study could have led to biases in the estimates of the impact of influenza in some age groups. Finally, although the current model was involved in some independent factors such as influenza activity proxy, absolute humidity, Spring Festival holiday, and containment against emerging COVID‐19, other unobserved factors including epidemics of other infectious diseases or other environmental factors as confounders could also affect the outpatient burden. For example, respiratory syncytial virus (RSV) and influenza are thought to have the most impact among all respiratory viruses. RSV seasons may overlap with influenza epidemics. Due to the lack of surveillance data of other respiratory viruses, we might overestimate the burden of influenza to a certain extent.

In conclusion, we found considerable outpatient burden attributable to influenza in all ages in Ningbo from 2017–2018 to 2019–2020. Influenza B/Yamagata virus had the most impact on the outpatient burden, compared with the other influenza subtypes or lineages. Children 0–4 and 5–14 years of age had the highest outpatient burden attributable to influenza during the study period. The influenza‐associated outpatient burden in people aged ≥60 years was higher than that in adults under 60 years. Although the wintertime influenza season in 2019–2020 ended earlier, we still identified substantial incidence rate of influenza infection indicating serology in people aged ≥60 years in the community with low influenza vaccination rate in this year. It is important to increase influenza vaccine coverage among high‐risk population including elderly people to reduce their risks of hospitalization and severe complications following infection because there are high risks of influenza infection and occurrence of clinical illness.

## AUTHOR CONTRIBUTIONS

**Cuiling Xu:** Conceptualization; data curation; investigation; methodology. **Xuying Lao:** Investigation; resources; validation. **Hongyu Li:** Investigation. **Libo Dong:** Methodology; validation. **Shumei Zou:** Investigation. **Yi Chen:** Investigation; validation. **Yongquan Gu:** Project administration; resources. **Yueqin Zhu:** Investigation; project administration. **Pingfeng Xuan:** Investigation. **Weijuan Huang:** Methodology; supervision. **Dayan Wang:** Funding acquisition; methodology; supervision. **Bo Yi:** Funding acquisition; methodology; project administration; resources; supervision.

### PEER REVIEW

The peer review history for this article is available at https://publons.com/publon/10.1111/irv.12935.

## Data Availability

The datasets in our study are available from the first author and correspondence author.
